# Severe Acute Liver Dysfunction Induces Delayed Hepatocyte Swelling and Cytoplasmic Vacuolization, and Delayed Cortical Neuronal Cell Death

**DOI:** 10.3390/ijms24087351

**Published:** 2023-04-16

**Authors:** Kazuhiko Nakadate, Chiaki Sono, Homura Mita, Yuki Itakura, Kiyoharu Kawakami

**Affiliations:** Department of Basic Science, Educational and Research Center for Pharmacy, Meiji Pharmaceutical University, 2-522-1 Noshio, Kiyose 204-8588, Tokyo, Japan

**Keywords:** brain edema, astrocyte, pathology, hepatic encephalopathy, transmission electron microscope, scanning electron microscope, iNOS, microglial cell

## Abstract

Liver dysfunction is the main cause of hepatic encephalopathy. However, histopathological changes in the brain associated with hepatic encephalopathy remain unclear. Therefore, we investigated pathological changes in the liver and brain using an acute hepatic encephalopathy mouse model. After administering ammonium acetate, a transient increase in the blood ammonia level was observed, which returned to normal levels after 24 h. Consciousness and motor levels also returned to normal. It was revealed that hepatocyte swelling, and cytoplasmic vacuolization progressed over time in the liver tissue. Blood biochemistry also suggested hepatocyte dysfunction. In the brain, histopathological changes, such as perivascular astrocyte swelling, were observed 3 h after ammonium acetate administration. Abnormalities in neuronal organelles, especially mitochondria and rough endoplasmic reticulum, were also observed. Additionally, neuronal cell death was observed 24 h post-ammonia treatment when blood ammonia levels had returned to normal. Activation of reactive microglia and increased expression of inducible nitric oxide synthase (iNOS) were also observed seven days after a transient increase in blood ammonia. These results suggest that delayed neuronal atrophy could be iNOS-mediated cell death due to activation of reactive microglia. The findings also suggest that severe acute hepatic encephalopathy causes continued delayed brain cytotoxicity even after consciousness recovery.

## 1. Introduction

The liver is a metabolic organ involved in detoxification, nutritional metabolism, blood volume maintenance, and hormone regulation [1]. Hepatic encephalopathy (HE) is the leading cause of liver failure and is frequently associated with the progression of end-stage liver disease [2]. Furthermore, liver failure is closely associated with the development of intracranial hypertension, which clinically manifests as cerebral edema and has been suggested to play a crucial role in HE development. HE ranges from minimal dysfunction to coma, resulting from ammonia-related neurotoxicity, where metabolic dysfunction causes glutamine accumulation, astrocyte swelling, and nitric oxide-induced vasodilation [3]. Increased intracranial pressure, secondary to cerebral edema, is common in patients with liver failure and occurs in 80% of comatose patients [3,4,5]. In patients with acute HE, cerebral edema plays an important role in the associated neurological deterioration. Increased fluid infiltration from blood vessels into the brain increases brain volume and intracranial pressure [2]. Vasogenic edema is primarily caused by the disruption of tight endothelial junctions that make up the blood–brain barrier (BBB) [6]. Perturbation of cell metabolism impairs the sodium and potassium pumps’ function in glial membranes surrounding the blood vessels, leading to the accumulation of osmotically active molecules and retention of sodium and water in cells, thus, resulting in swollen glial cells and neuronal cytotoxic edema [6,7,8]. The molecular mechanisms underlying glial cell swelling have not been fully elucidated. However, they are thought to be associated with osmo- or stretch-sensitive intracellular signaling cascades involving [Ca^2+^]-ion transients, aquaporins (AQPs, primarily Aquaporin4), and volume-regulated anion channels [8,9,10,11]. When glial cell swelling occurs, neuronal atrophy is triggered in the brain. In HE, the primary target of the glial cells affected in the brain are the perivascular astrocytes (glial fibrillary acidic protein; GFAP positive cells), but it is thought that microglia (ionized calcium-binding adapter molecule 1; Iba1 positive cells) that display immune responses are also affected. Accompanied by an immune response, a nitric oxide-producing enzyme that can be induced by cytokines (inducible nitric oxide synthase: iNOS) and inflammatory cytokines (interleukin [IL]-1β, IL-18, CD16, and tumor necrosis factor-α [TNF-α]) play a role in inflammation [12,13]. However, the presence or absence of glial cell degeneration and inflammatory response in the brain in HE has not yet been clarified in detail.

In HE research, few basic studies have investigated pathological changes in the liver, except for reports in the clinical pathology field. Even in such settings, there are some reports on acetaminophen-induced acute liver failure [14]. In this study, N-acetylcysteine had a hepatoprotective effect and prevented neurological complications, such as HE and cerebral edema. Similarly, there is a report on the effect of dihydromyricetin in acute liver failure by immunohistological analysis of liver tissue changes and brain astrocytes [15].

Several studies have reported the mechanism of action and related receptors for cerebral edema in patients with HE. However, there are no reports on a concurrent histopathological and ultrastructural analysis of liver injury and cerebral edema. Furthermore, the cause of late-onset cerebral dysfunction has not been reported, and no time-course analysis has been performed. Therefore, the relationship between brain pathology and pathological changes in the liver remains unclear. This study aimed to use light and electron microscopy for pathological and ultrastructural analysis of the brain and liver in HE. In addition, we performed biochemical data analysis and Western blot analysis to evaluate delayed brain dysfunction after acute HE.

## 2. Results

### 2.1. Induction of Hepatic Encephalopathy

**Figure 1:**
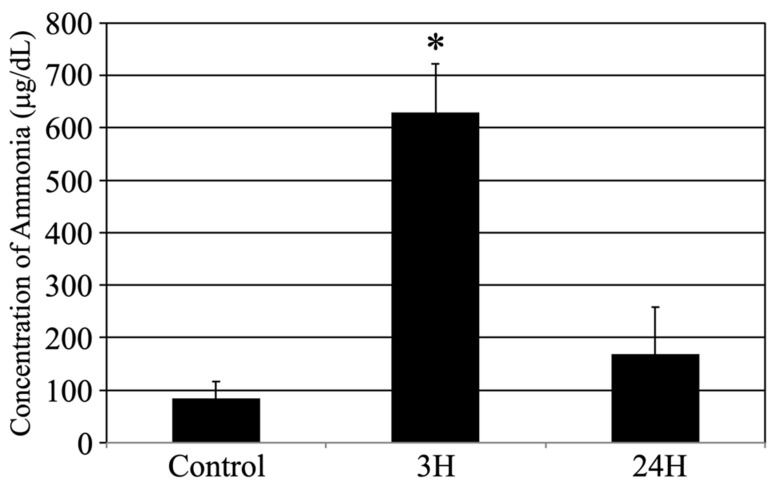
Control, 3H, and 24H indicate the average blood ammonia concentration for the animals in the vehicle treatment control group and the treated animal at 3 and 24 h after ammonia treatment, respectively. Data are expressed as the mean ± standard error. *: *p* < 0.05 compared with the control value.

This study was designed to investigate whether acute hepatic dysfunction affects the brain. First, the sequential changes in the blood ammonia concentration in each animal following treatment with the ammonia-inducing compound (Figure 1) were evaluated. The average blood ammonia value recorded was low for the control animals (84.2 ± 31.9 μg/dL) and high for the treated animals at 3 h post-injection (629.6 ± 92.5 μg/dL, *p* < 0.05) before almost returning to control levels at 24 h post-injection (169.3 ± 89.3 μg/dL).

### 2.2. Histopathological Evaluation of the Hepatic Structural Changes in Response to Hepatic Encephalopathy

**Figure 2:**
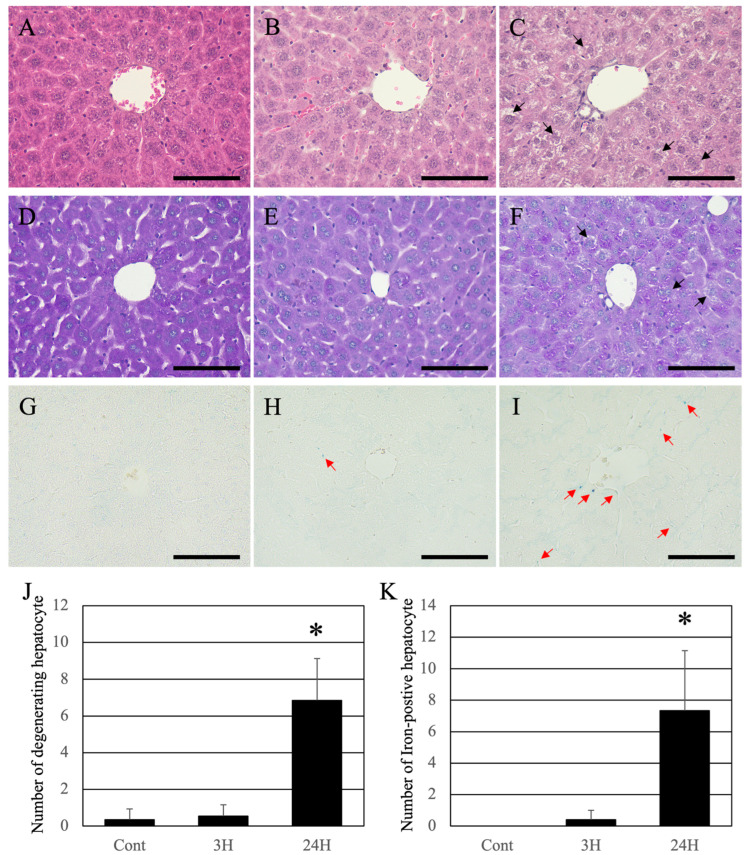
Control (**A**,**D**,**G**), 3H (**B**,**E**,**H**), and 24H (**C**,**F**,**I**) representative images of the hepatic tissues from the control animals and those from animals sacrificed at 3 and 24 h post-ammonia treatment, respectively. (**A**–**C**) are high-magnification views of the hematoxylin-eosin (HE) stained hepatic tissues, while (**D**–**F**) are high-magnification views of the Periodic acid–Schiff (PAS) stained hepatic tissues. (**G**–**I**) are high-magnification views of the Iron-stained hepatic tissues. Black arrows indicate the hepatocytes exhibiting swelling and/or cytoplasmic vacuolization. Red arrows indicate the iron-positive sites. All scale bars = 100 μm. (**J**) shows the number of hepatocytes exhibiting swelling and/or cytoplasmic vacuolization. (**K**) shows the number of iron-positive hepatocytes. Data are expressed as mean ± standard deviation. * *p* < 0.05, compared to the control.

Histopathological evaluation of the liver was performed to evaluate the relationship between pathological changes in the brain and liver (Figure 2). Using the HE staining method, the livers of the control animals were found to be healthy, with normal hepatocytes and sinusoids (Figure 2A). These tissues were also stained with uniform eosin. In both the ammonia treatment groups, eosin staining was significantly reduced (eosinophilic changes: Figure 2B,C). In addition, many hepatocytes exhibited vacuolization and/or swelling, indicating cell death at 24 h after ammonia treatment (arrows in Figure 2C). For quantification analysis, we counted the number of hepatocytes exhibiting swelling and/or cytoplasmic vacuolization; the number of such hepatocytes in the control and 3 h post-ammonia treatment groups was very low. However, in the 24 h post-ammonia treatment group, the number of hepatocytes exhibiting swelling and/or cytoplasmic vacuolization was significantly increased (Figure 2J).

Periodic acid–Schiff (PAS) staining was normal in control livers (Figure 2D). Like HE, a decrease in PAS staining was observed in both ammonia-treated groups; however, intrahepatic fibrosis did not occur (Figure 2E,F). In the animal’s liver of the 24 h post-treatment group, uneven staining was observed in hepatocytes, indicating that the hepatocytes were damaged (arrows in Figure 2F). As hemosiderin deposition (iron deposition) occurs with continued liver injury, iron staining was performed (Berlin-blue iron staining). Normal liver tissue was negative for hemosiderin (Figure 2G). A low number of iron positives hepatocytes was detected in the livers of ammonia-treated animals (3 h post-treatment) (red arrows in Figure 2H). In contrast, several iron-positive sites were seen in the animals 24 h post-ammonia treatment (red arrows in Figure 2I). For quantification analysis, we counted the iron-positive hepatocytes. The number of iron-positive hepatocytes in the control and 3 h post-ammonia treatment groups was very low. However, in the 24 h post-ammonia treatment group, the number of iron-positive hepatocytes was significantly increased (Figure 2K).

**Figure 3:**
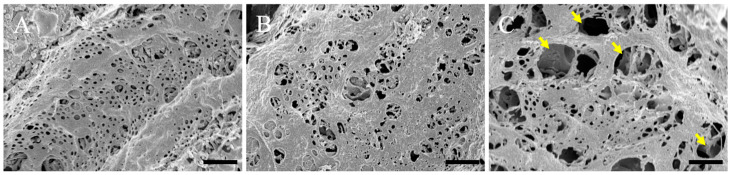
Scanning electron microscopy micrographs of the sinusoidal endothelium are shown. The sinusoidal endothelium of the control is shown (**A**). (**B**,**C**) Sinusoidal endothelium of animals, 3 h and 24 h post-ammonia treatment, respectively. Yellow arrows show the swelling of sinusoidal fenestrations. All scale bars = 1 µm.

Using scanning electron microscopy, we examined the pathological changes in the hepatic sinusoid. The sinusoidal endothelium of control (normal) mice showed a healthy structure (Figure 3A), while the ultrastructure of the sinusoidal endothelium of the 3 h post-ammonia treatment mice showed moderate dilatation of the sinusoidal capillaries (Figure 3B). The ultrastructure of the sinusoidal endothelium of the 24 h post-ammonia treatment mice showed significant dilatation of the sinusoidal capillaries (Figure 3C) and swelling of sinusoidal fenestrations (yellow arrows in Figure 3C).

### 2.3. Biochemical Analyses

Various biochemical parameters of the different treatment groups were evaluated using commercial kits (Table 1). These evaluations revealed that the total protein (TP) content was significantly decreased while aspartate aminotransferase (AST) significantly increased at 3 h post-ammonia treatment compared to the control group. In addition, evaluations of the 24 h post-ammonia treatment animals revealed that while TP returned to control levels, AST levels remained elevated. In contrast, alanine aminotransferase (ALT) levels were significantly increased, while both albumin (ALB) and alkaline phosphatase (ALP) values showed an increasing trend, but no significant change was observed.

### 2.4. Histopathological Evaluation of the Changes in the Cerebral Cortex in Response to Hepatic Encephalopathy

**Figure 4:**
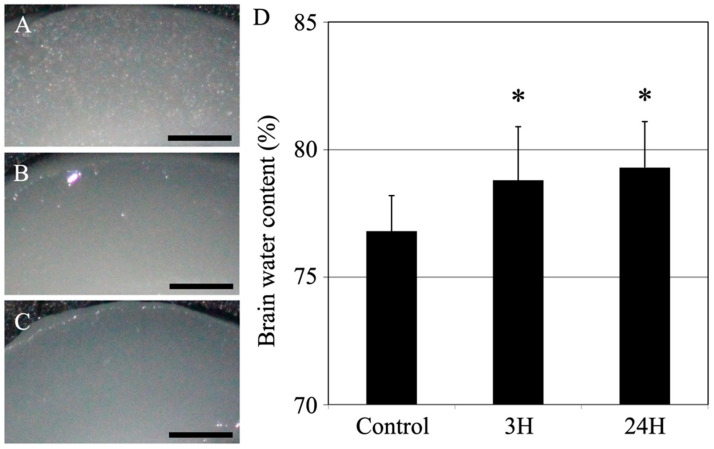
Control (**A**), 3H (**B**), and 24H (**C**) representative images of the cerebral cortical tissues from the control animals and those from animals sacrificed at 3 h and 24 h after ammonia treatment, respectively. All scale bars = 500 μm. (**D**) Control, 3H, and 24H indicate the brain water contents for the animals in the control group and the treated animal at 3 and 24 h after ammonia treatment, respectively. Data are expressed as the mean ± standard deviation. *: *p* < 0.05 compared with the control value.

Histopathological evaluation of the changes in the cerebral cortices was conducted among animals with encephalopathy. First, we examined the water content in the cerebral cortex (Figure 4). Compared to the control mice (76.8 ± 1.4%), the treated animals showed significantly increased water content in the cerebral cortex (78.8 ± 2.1%; *p* < 0.05) 3 h after ammonia treatment. Similarly, the cerebral cortex’s water content in the 24 h post-treatment animals showed a significant increase compared to the control mice (79.3 ± 1.8%; *p* < 0.05).

**Figure 5:**
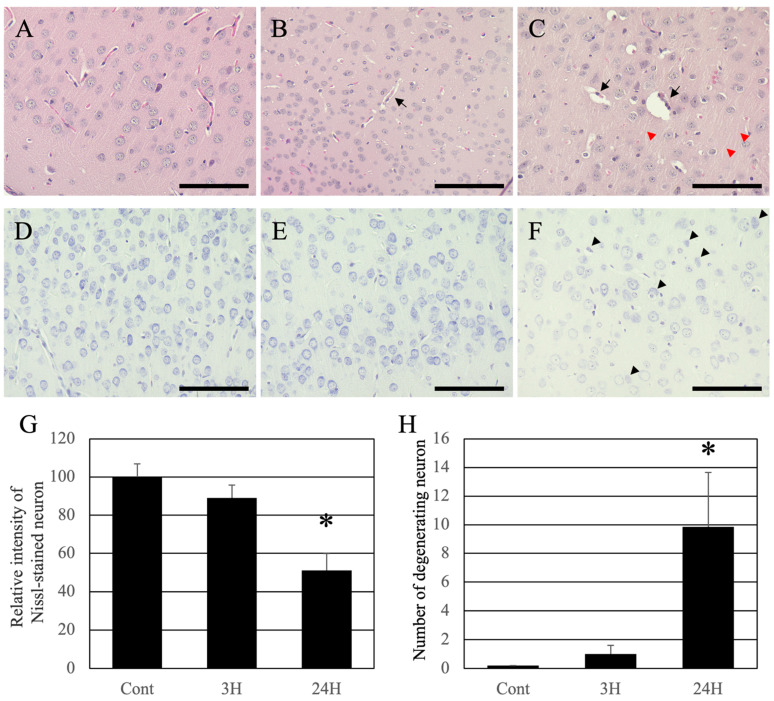
Control (**A**,**D**), 3H (**B**,**E**), and 24H (**C**,**F**) representative optical images of the cerebral cortical tissues from the control animals and those from animals sacrificed at 3 h and 24 h after ammonia treatment, respectively. (**A**–**C**) are high-magnification views of the hematoxylin-eosin-stained cerebral cortical tissues. (**D**–**F**) are high-magnification views of the Nissl-stained cerebral cortical tissues. The black arrows indicate perivascular edema of the cerebral cortex layer II/III. Red arrowheads indicate the swollen fibers of the cerebral cortex layer II/III. Black arrowheads indicate the neuronal atrophy of the cerebral cortex layer II/III. All scale bars = 100 μm. (**G**) shows the relative intensity of Nissl-stained neurons. Data are shown as relative intensity (100 = the intensity of the control value). (**H**) shows the number of atrophying neurons. Data are expressed as mean ± standard deviation. *: *p* < 0.05 compared with the control value.

Histopathological changes in the cerebral cortex were evaluated using hematoxylin-eosin (HE) staining (Figure 5), and it revealed that the cortical tissues of the control animals were healthy, and the neurons and blood vessels were normal (Figure 5A). On the contrary, eosin staining was reduced in the cortical tissues of both ammonia treatment groups (Figure 5B,C), and the perivascular spaces were swollen (arrows in Figure 5B,C). Twenty-four hours post-ammonia administration, the nerve fibers were swollen (red arrowheads in Figure 5C).

Nissl staining was performed to identify pathological changes in neuronal cell bodies. The cortical neurons of normal animals were normal (Figure 5D). The dyeing property of Nissl decreased slightly 3 h post-administration and remarkably decreased 24 h post-administration (Figure 5E,F). For quantification analysis, the intensity of Nissl-stained neurons was measured (Figure 5G). The intensity of Nissl-stained neurons in the control and 3 h post-ammonia treatment groups was very high. However, in the 24 h post-ammonia treatment group, the number of atrophying neurons was significantly decreased. Moreover, many atrophying neurons in the cerebral cortex were identified (black arrowheads in Figure 5F). For quantification analysis, we counted the number of atrophying neurons, which were very low in the control and 3 h post-ammonia treatment groups. However, in the 24 h post-ammonia treatment group, the number of atrophying neurons was significantly increased (Figure 5H).

**Figure 6:**
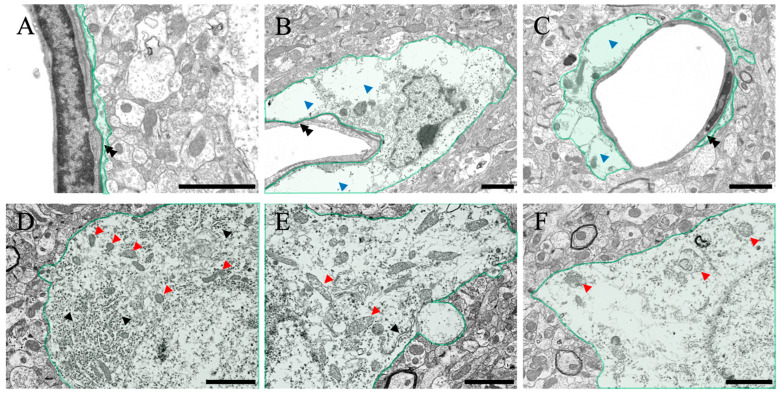
Control (**A**,**D**), 3H (**B**,**E**), and 24H (**C**,**F**) representative electron microscopy images of the cerebral cortical tissues from the control animals and those from animals sacrificed 3 h and 24 h after ammonia treatment, respectively. (**A**–**C**) show the blood vessel and perivascular astrocytes in the cerebral cortex (green color). (**D**–**F**) show the neurons in the cerebral cortex (green color). Black double arrowheads show the basal membrane. Blue arrowheads indicate the perivascular edema of the cerebral cortex layer II/III. Black arrowheads indicate the rough endoplasmic reticulum. Red arrowheads indicate mitochondria. All scale bars = 2 μm.

The structural changes observed in the optical microscope were further examined in detail using a transmission electron microscope (Figure 6). First, regarding perivascular pathological changes, perivascular edema was observed in both ammonia-treated groups compared with the control group (blue arrowheads in Figure 6B,C). However, no damage was observed in the basal membrane (black double arrowheads in Figure 6A–C) or vascular endothelial cells. Second, regarding pathological changes in neuronal cell bodies of neurons, many intracellular organelles, such as the rough endoplasmic reticulum (black arrowheads in Figure 6D) and mitochondria (red arrowheads in Figure 6D), were confirmed in normal neurons (Figure 6D). A decrease in the rough endoplasmic reticulum (black arrowheads in Figure 6E) and degeneration of mitochondria (red arrowheads in Figure 6E,F) were observed in both ammonia-treated groups (Figure 6E,F).

**Figure 7:**
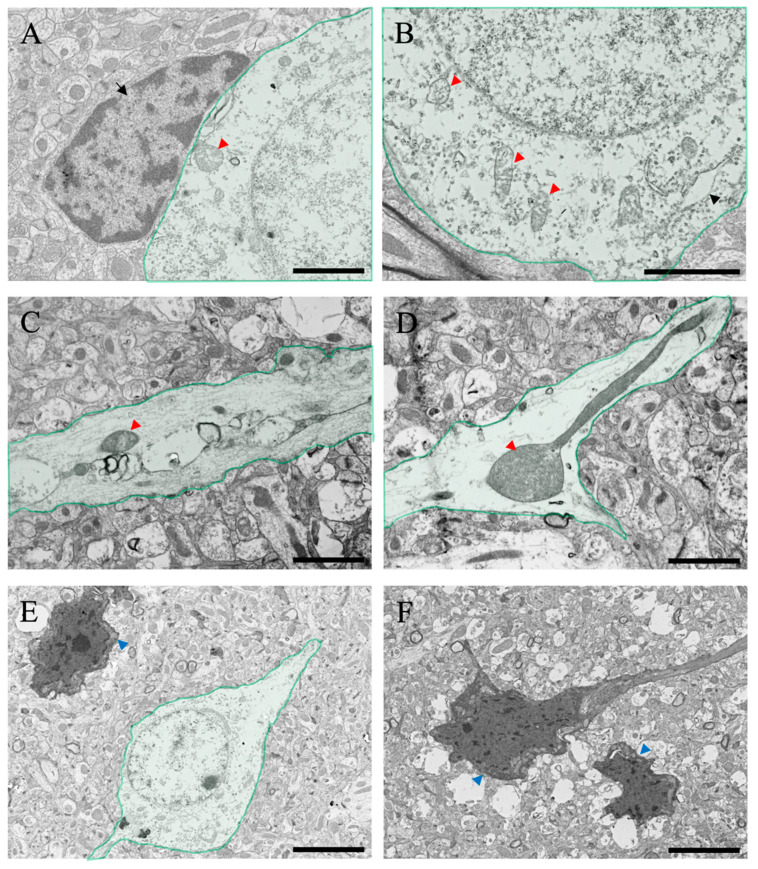
(**A**–**F**) Transmitted electron microscopic images of the cerebral cortical tissues from the ammonia-treated animals 24 h post-administration. Green-colored neurons in the cerebral cortex are shown. Black arrow in (**A**) indicates the intact perineuronal glial cell (astrocyte). Black arrowheads indicate the rough endoplasmic reticulum. Red arrowheads indicate degenerating mitochondria. Blue arrowheads indicate neuronal atrophy. Scale bars in (**A**–**D**) = 2 μm, and (**E**,**F**) = 5 μm.

After ammonia administration, various degenerative findings were observed in the cerebral cortex of the 24 h group (Figure 7). Perineural glial cells (astrocytes), with reduced organelles and mitochondrial degeneration (red arrowheads in Figure 7A,B), were observed (arrow in Figure 7A). Swelling of nerve fibers was confirmed through optical microscopy, but similar swelling was also confirmed via electron microscopic analysis (Figure 7C,D), and degeneration of mitochondria within the fibers was also observed (red arrowheads in Figure 7C,D). In addition, several neuronal cell deaths were observed (blue arrowheads in Figure 7E,F), which were considered to be caused due to ammonia’s toxic effect. In addition, several large vacuoles and edema spaces were observed around neuronal cell death sites, suggesting that damaged astrocytes surrounding neurons may cause neuronal cell death.

### 2.5. Brain Inflammatory Response after Hepatic Encephalopathy

**Figure 8:**
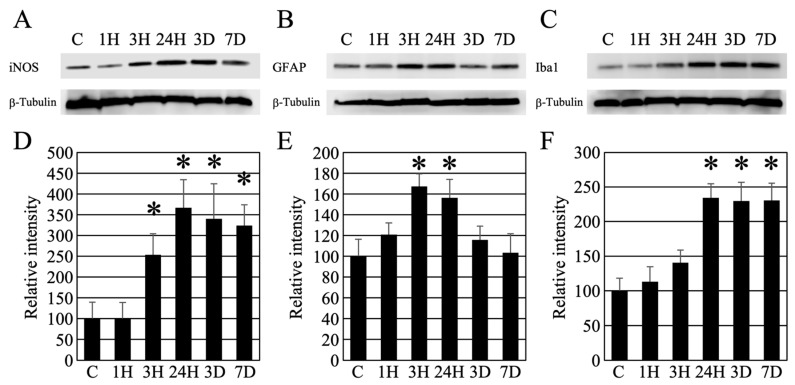
(**A**–**C**) Western blot analysis revealed the timing of iNOS, GFAP, and Iba1 expression following hepatic encephalopathy. In addition, as an internal control, the expression level of β-tubulin is presented. (**D**–**F**) Densitometry analysis of the Western blot data (each number = 5). Data are expressed as mean ± standard deviation. Data are shown as relative intensity (100 = the intensity of the control value). C, 1H, 3H, 24H, 3D, and 7D indicate the various treatment groups post-ammonia treatment (control, 1 h, 3 h, 24 h, 3 days, and 7 days), respectively. *: *p* < 0.05 compared with the control.

Western blotting was used to examine whether neuronal atrophy in the brain was due to inflammatory reactions. First, iNOS, which is related to neuronal atrophy, was examined for changes from the onset of HE (Figure 8A). The optical density of the bands increased over time and remained significant even after seven days (Figure 8D). Since perivascular astrocyte swelling was remarkable in HE, we decided to investigate the astrocytes (Figure 8B). Using an anti-GFAP antibody (astrocyte marker) [16], we found that the amount of GFAP peaked at 3 h to 24 h after induction of HE and showed a transient increase, after which there was no significant difference from the control value (Figure 8B,E). As glial cells cause inflammation of nerve cells, we then examined microglia using anti-Iba1 antibody (microglial marker) (Figure 8C) [17]. The amount of Iba1 protein showed a pattern similar to that of iNOS expression. In summary, the optical density of the bands increased over time and remained significant even after seven days (Figure 8F).

## 3. Discussion

Ammonia is normally produced in the gastrointestinal tract via the breakdown of proteins and amino acids by host and bacterial enzymes. Ammonia then enters the portal circulation and is metabolized in the liver, urea and glutamine before being excreted. When the liver malfunctions, blood ammonia levels increase, leading to an influx of ammonia into astrocytes, thus increasing their volume and inducing cerebral edema [18]. The normal human blood ammonia concentration is 30–80 μg/dL (9–35 μmol/L), and the reference range for blood ammonia concentration decreases with age [19,20]. Arterial ammonia concentrations above 340 μg/dL (200 μmol/L) increase the risk of cerebral edema and coma [19,20]. Individuals with elevated blood ammonia levels often present as comatose patients or patients with cerebral edema [20]. Previous acute-phase studies using animals show a transient increase in blood ammonia levels after ammonia administration [21,22]. Urea metabolism and urinary excretion also increase with increased blood ammonia concentration [22]. This metabolism is triggered in parallel with blood ammonia levels, as well as reactions during food intake [23]. In this study, blood ammonia concentration increased transiently, and mice fell into a coma; however, the mice woke up from the coma in a few hours with recovered motor function. Urinary metabolism is also considered a factor in the relationship between the coma state and blood ammonia level.

Several methods have been devised to study HE. Based on previous reports using ammonium acetate [24,25,26,27,28], a simple and accurate animal model was devised to exhibit elevated blood ammonia levels. Our mouse model was used to pathologically investigate how elevated blood ammonia levels affect the brain. In this study, after two episodes of ammonia treatment, the mice fell into a coma for several hours; however, after the ammonia treatment, the mice woke up within 6 h, returned to normal, and exhibited motor function without a single death. This conditional change in mice correlated with transient increases in blood ammonia levels (Figure 1). These treatments induced a transient decrease in the total protein levels, which returned to normal after 24 h. (Table 1). In addition, ALB levels did not change. Therefore, these animals may have maintained blood osmolarity. Furthermore, as we have previously reported [29], the osmotic pressure of the reagents and fixatives used in this study is within the normal range, indicating that the pathological changes are not due to side effects.

Histological analysis of the liver (Figure 2) and biochemical analysis of the blood (Table 1) were performed in the same individuals. Both HE and PAS staining, and hemosiderin reactions were normal in the normal liver. Conversely, ammonia administration significantly decreased eosin staining in the HE-stained images. This result suggests that the cytoplasm of hepatocytes was damaged. A remarkable image was also found in individuals of 24 h post-ammonia treatment, suggesting that hepatocytes exhibiting vacuolization and swelling had progressed. Although the PAS-stained image showed a decrease in stainability, liver fibrosis was not observed. Furthermore, despite many positive structures in the hemosiderin reaction, no significant iron accumulation was observed, and the degeneration findings were not dramatic. This correlates with the significant increase in liver marker values in the blood biochemical data of 24 h animals group after the blood ammonia concentration decreased. Furthermore, scanning electron microscopy analysis results also indicate that sinusoids are gradually damaged (Figure 3), suggesting that delayed hepatocyte deterioration is induced during recovery after acute HE.

In addition to the liver analysis, macroscopic analysis of the cerebral cortex (water content measurement) showed a significant increase in water content in the ammonia-treated group, indicating the presence of cerebral edema (Figure 4). Similarly, HE staining (Figure 5) also revealed cerebral edema in these animals, which included swelling of the tissue around the blood vessels in the cerebral cortex. This result is consistent with that of a previous study [20]. The HE-stained images of the cerebral cortex, taken 24 h post-ammonia treatment, showed persistent perivascular swelling, consistent with the results of water content measurements. Furthermore, the microscopic analysis using a transmission electron microscope (Figure 6 and Figure 7) showed swelling around blood vessels and astrocytes for the ammonia group. Previous studies on HE have reported swelling around the blood vessels that make up the BBB of the brain’s cerebral cortex. AQP4 is closely associated with astrocytic activity in these vessels [30,31,32,33,34,35,36]. This study detected similar swelling around blood vessels in the brain’s cerebral cortex following ammonium acetate administration, suggesting swelling due to AQP4. Aquaporin4 (AQP4), found in the brain, is a bidirectional transmembrane water channel specifically localized in the terminal feet of astrocytes. AQP4 may play an important role in the exacerbation and resolution of traumatic cytotoxic brain edema. Physiologically, AQP4 associates with the inwardly rectifying K^+^ channel Kir4.1 and colocalizes with AQP4 on the terminal feet of rodent astrocytes. Thus, it clears water via AQP4 and K^+^ via Kir4.1, with high neuronal activity from the extracellular compartment to neighboring astrocytes [37]. In pathophysiological situations, AQP4 alone also transports water across cell membranes in both directions along osmotic gradients [38]. Deletion of AQP4 has been reported to reduce cerebral edema [31]. Many studies have suggested that perivascular astrocyte swelling is mediated by astrocytic AQP4 in HE. Various studies have been conducted on how ammonia affects astrocytes through various mechanisms, one of which suggests that AQP4 acts as an ammonia channel, implying that blood ammonia directly enters the astrocytes through AQP4 [39]. Glutamine is produced in the presence of glutamine synthetase from ammonia taken up by astrocytes. A large amount of glutamic acid is taken up by neurons, disturbing the neurotransmitter balance, and thus inducing neuronal dysfunction. It has been suggested that mitochondrial dysfunction in neurons, caused by altered neurotransmission alone, may lead to neuronal atrophy [40,41].

The Nissl staining results confirmed a significant decrease in nerve cell staining after ammonia administration, especially in treated animals 24 h later (Figure 5). Nissl staining strongly stained the rough endoplasmic reticulum in the nerve cell body, suggesting a decrease in the rough endoplasmic reticulum in the nerve cell body of post-treated animals (24 h post-treatment). Therefore, microscopic analysis was performed using a transmission electron microscope (Figure 6 and Figure 7). As a result, a gradual decrease in rough endoplasmic reticulum was confirmed at 3 h post-treatment of ammonia, and a marked decrease was observed at 24 h. Furthermore, several abnormal mitochondria were observed, suggesting that nerve cells were greatly affected. The ultrafine morphological observations confirmed various aspects of nerve cells in animals 24 h after ammonia administration (Figure 7). Abnormal mitochondria were observed not only in the nerve cell bodies but also in the neurites. The mitochondrial permeability transition is a Ca^2+^-dependent process characterized by the opening of permeability transition pores present in the inner mitochondrial membrane. Opening the pore results in increased permeability to protons, ions, and other solutes of ≤1500 Da, leading to a collapse of the mitochondrial inner membrane potential, ultimately resulting in decreased oxidative phosphorylation and bioenergetic failure [42]. The induction of mitochondrial permeability transition can also lead to secondary oxidative stress [43,44]. It has been reported that the administration of ammonia in cultured astrocytes induces mitochondrial permeability transition [45]. The mitochondrial permeability transition was also induced in the HE rat model [46], suggesting that the results of the observation of abnormal mitochondria in this study confirmed the biochemical data obtained using ultra-microstructure analysis. Moreover, ammonia-induced mitochondrial dysfunction has been reported to affect brain energy metabolism [47,48,49]. The reduction and abnormal morphology of the mitochondria in neurons that we confirmed in this study suggest the possibility that the energy metabolism of neurons themselves is triggered. In addition, many cells undergo degeneration and cell death. Several vacuoles and edematous spaces were found around dead neurons, and most astrocytes around the blood vessels were swollen in ammonia-treated groups. In contrast, astrocytes around live neurons showed a normal structure in animals 24 h after ammonia administration. It remains to be determined whether this result is due to the distance from the blood vessels, the astrocytes around the blood vessels exposed to high concentrations of ammonia, and the distant location of astrocytes, or whether it is due to functional differences in astrocytes [16,50,51,52,53]. Therefore, it is necessary to consider this issue in future studies.

Brain inflammation is known to be involved in neuronal atrophy. Inflammatory cytokines (iNOS, IL-1β, IL-18, CD16, and TNF-α) play a role in inflammation [12,13]. We investigated the possibility that inflammatory cytokines caused delayed neuronal atrophy after the decrease in blood ammonia level observed in this study (Figure 8). We analyzed the expression changes of iNOS, which is thought to be strongly involved in neuronal atrophy. A further increase in iNOS expression was confirmed 24 h after the decrease in blood ammonia level, and the increased expression was maintained even after seven days. This result suggests that iNOS is involved in delayed neuronal atrophy. We also investigated the activation of astrocytes to examine the relationship with iNOS. After induction of HE, GFAP expression transiently peaked at 3 h to 24 h, but then decreased sharply and returned to normal values. In addition, we observed the Iba1 protein (microglial marker), which peaked 24 h after the induction of HE, and the increased expression was maintained even after seven days. These results suggest that HE first triggers swelling of astrocytes in the brain, followed by activation of reactive microglia, which consequently increases iNOS expression. These morphological changes and increased expression of various inflammatory cytokines are thought to induce delayed cytopathic atrophy in neurons. On the other hand, it has been reported that there is no elevation of GFAP in the HE brain [54]. This difference is thought to be due to the blood ammonia concentration. It is believed that severe symptoms of high blood ammonia concentration have a profound effect on glial cells in the brain.

Coma and cerebral edema occur in almost all patients when blood ammonia levels increase as liver disease progresses, including HE [55,56,57,58]. HE is diagnosed comprehensively by distinguishing it from other diseases based on abnormal liver function; the presence or absence of a history of liver disease; neuropsychiatric symptoms, such as disturbance of consciousness, hyperammonemia, electroencephalogram abnormalities, and image inspection; and biochemical examination results. After the diagnosis of hepatic encephalopathy, removal of toxic substances, mainly ammonia, and correction of metabolism of amino acids, etc., are performed from an early stage. Thus, although human patients rarely reach the high levels of ammonia described in this study, an increasing number of patients with various liver dysfunctions, including HE, was reported [59,60,61,62,63,64]. Patients often present with pathological conditions, such as liver cirrhosis, and it is thought that in vivo histopathological examination of the liver is necessary. However, histopathological examination using liver biopsy is rarely performed at the time of examination. Therefore, it is increasingly important to understand and assess the precise pathological effects during the acute phase of the conditions on the liver and brain. The findings of this study are expected to contribute to therapeutic methods for delayed cell death in the brain and facilitate the introduction and development of early treatment methods. The results of the current study will serve as the basis for both basic research and clinical application.

## 4. Materials and Methods

### 4.1. Study Design

We used 78 C57BL/6J apparently healthy male mice (10 weeks old; Charles River, Yokohama, Japan). The mice were housed under temperature and humidity-controlled conditions with a 12:12 h light–dark cycle and free access to food and water. Animal experiments were performed following the National Institutes of Health Guide for the Care and Use of Laboratory Animals, and the protocol was approved by the Laboratory Animal Ethics Committee of Meiji Pharmaceutical University (No. 2704, 1 April 2017–2022). All efforts were made to minimize animal suffering and to reduce the number of animals used in the study.

### 4.2. Inducing Acute Liver Failure

According to our previous study [29], acute liver failure was induced in an animal model. Using this method, it is possible to raise blood ammonia levels to coma-inducing levels without killing the animal. Fifty-seven 10-week-old C57BL/6J mice were treated intraperitoneally with ammonium acetate (4.5 mmol/kg body weight, Sigma-Aldrich, St. Louis, MO, USA) using two intraperitoneal injections with a 15 min interval between injections. Twenty-one age-matched male mice were intraperitoneally injected with saline and used as controls.

### 4.3. Biochemical Analysis

Using an animal restrainer for laboratory mice (CL-4903, CLEA-Japan Co., Tokyo, Japan), blood samples were collected from the tail vein of the unanesthetized animals (10 mice per group) from all groups (3 h and 24 h after ammonium acetate or vehicle injection), and 100 μL of each sample was diluted in an equivalent volume of heparin-supplemented physiological saline. These samples were then centrifuged (1500× *g*, 10 min, room temperature), and the plasma obtained after centrifugation was quickly cryopreserved at −80 °C for evaluation. The plasma ammonia concentrations were determined using a Cica-liquid NH_3_ kit (Kanto Chemical Co., Inc., Tokyo, Japan). TP, ALB, AST, ALT, and ALP concentrations were also determined using the appropriate test kit (Wako Pure Chemical Industries, Ltd., Tokyo, Japan).

### 4.4. Tissue Preparation

Tissue preparation for histochemistry and electron microscopy was performed as previously described [65,66,67,68].

For optical microscopy, brain and liver tissues were collected from animals in the control and treatment groups (six mice per group). They were fixed with 4% paraformaldehyde in 0.1 M phosphate buffer (PB) (pH 7.4). The brains and medial lobe of the liver were sliced into 2 mm thick sections using a slicer. The sections were immersed in graded concentrations of ethanol, cleared with Lemosol A (Wako Pure Chemical Industries, Ltd., Tokyo, Japan), and embedded in paraffin.

For transmission electron microscopy, brain tissues were collected from animals in both control and treatment groups (4 mice per group). They were fixed with 4% paraformaldehyde and 1% glutaraldehyde in 0.1 M PB (pH 7.4). Brains were sliced into 1 mm thick sections using a slicer. Sections were immersed in osmium tetroxide (TAAB Laboratories, Ltd., Aldermaston, UK) for 2 h, dehydrated in ethanol, and embedded in Epon-812 resin (TAAB Laboratories, Ltd., Aldermaston, UK).

For scanning electron microscopy, the medial lobe of the liver tissue was collected from animals of both control and treated groups (4 mice per group). The livers from each group were fixed with 4% paraformaldehyde and 2% glutaraldehyde in 0.1 M PB (pH 7.4). After washing with PB and cutting, the liver blocks were immersed in osmium tetroxide solution, substituted with 50% DMSO, cracked in liquid nitrogen, dehydrated through graded concentrations of ethanol, and substituted with 2-Methyl-2-propanol. All the cracked blocks were freeze-dried and sputter-coated with gold.

### 4.5. Brain Water Measurement

Cerebral cortices from each group (6 mice per group) were quickly cut into 2 mm thick slices, flash-frozen in liquid nitrogen, and stored at −70 °C for 48 h. Water content was measured gravimetrically using a density gradient of bromobenzene–kerosene (Fisher Scientific, Pittsburgh, PA, USA) pre-calibrated with potassium sulfate (K_2_SO_4_) as previously described [14,69]. Cortical sections were placed on the fluid column, and the equilibration point was measured after 2 min. Four to five measurements were performed per animal, and the values were arithmetically averaged. The specific gravity of the tissue was calculated, and the results were expressed as a percentage of water content.

### 4.6. Histological Analysis

All staining required ultratome sectioning of paraffin-embedded brain and liver blocks. They were cut into 5 μm thick sections using a sliding microtome (REM-710; Yamato Kohki Industrial, Tokyo, Japan). All sections were mounted on glass slides, deparaffinized with Lemosol A immersed in graded concentrations of ethanol and distilled water.

For HE staining, sections of the brains and livers were stained with hematoxylin and eosin solutions (Muto Pure Chemicals Co., Ltd., Tokyo, Japan). After washing, the sections were dehydrated using graded concentrations of ethanol and Lemosol A, and a cover slip was placed.

For Nissl staining, the brain sections were stained with 0.1% cresyl violet solution (Abcam, Cambridge, UK). After washing, the sections were dehydrated using graded concentrations of ethanol, Lemosol A, and cover-slipped.

For PAS staining, liver sections were stained using a PAS solution kit (Muto pure chemicals Co., LTD., Tokyo, Japan). After washing, the sections were dehydrated using graded concentrations of ethanol, Lemosol A, and cover-slipped.

For iron staining (Berlin-blue iron staining), the liver sections were stained using an iron staining kit (Muto Pure Chemicals Co., Ltd., Tokyo, Japan). After washing, the sections were dehydrated using graded concentrations of ethanol, Lemosol A, and cover-slipped.

All section images were captured using a CCD camera (BZ-X700; Keyence, Japan).

Using HE-stained liver sections, the hepatocytes swelling and/or cytoplasmic vacuolization were counted (5 photos [500 μm × 500 μm]/3 animals/each group). We used iron-stained liver sections to count the iron-positive hepatocytes (5 photos [500 μm × 500 μm]/3 animals/each group). In addition, using Nissl-stained sections of the cerebral cortex, the intensity of Nissl-stained neurons was measured (40 neurons/5 photos [500 μm × 500 μm]/3 animals/each group). We also used the Nissl-stained sections of the cerebral cortex to count the neuronal atrophy (5 photos (500 μm × 500 μm)/3 animals/each group).

### 4.7. Electron Microscopical Analysis

For transmission electron microscopy, ultrathin sections (70 nm thick) were cut using a Leica EM UC6 Ultramicrotome (Leica Microsystems, Wetzlar, Germany) and placed onto grids (Veco, Eerbeek, The Netherlands). The electron-stained ultrathin sections were examined using a transmission electron microscope (HT7800, Hitachi, Tokyo, Japan), and images were captured using a CCD camera.

For scanning electron microscopy, sputter-coated liver blocks were examined under a scanning electron microscope (S-4700, Hitachi High-Technologies Corporation, Tokyo, Japan). Hepatic sinusoidal structures of control and ammonia-treated mice were randomly taken from the periportal to the centrilobular fenestrae.

### 4.8. Western Blot Analysis

As described previously [29,65,70], Western blot analysis was performed. Each of the five mice in each group (control, 1 h, 3 h, 24 h, 3 days, and 7 days after ammonium acetate injection) was used. Their brains were rapidly removed and homogenized in ice-cold homogenate buffer (20 mM Tris-HCl at pH 7.5, 1 mM ethylenediaminetetraacetic acid, 1 mM dithiothreitol, and 150 mM NaCl) supplemented with protease inhibitors (1 tablet/10 mL homogenate buffer, Complete^TM^ Mini, Roche Diagnostics, Basel, Switzerland). Homogenates were centrifuged at 500× *g* for 5 min at 4 °C, and the supernatants were placed in sodium dodecyl sulfate (SDS) sample buffer (62.5 mM Tris-HCl, pH 6.8, containing 3% SDS, 5% glycerol, and 2% 2-mercaptoethanol) and boiled for 5 min. Protein concentrations were evaluated using a protein assay kit (Bio-Rad Laboratories, Inc., Hercules, CA, USA).

Equal concentrations of protein from each group were then subjected to 7.5%, 10%, or 15% SDS-polyacrylamide gel electrophoresis (ATTO Corporation, Tokyo, Japan) and transferred onto a polyvinylidene difluoride membrane (ATTO Corporation, Tokyo, Japan). These membranes were blocked using a 10% blocking solution (ATTO Corporation, Tokyo, Japan) in phosphate-buffered saline (PBS) containing 0.1% Tween 20 (TPBS), for 1 h at room temperature (RT). The membranes were then incubated with rabbit anti-GFAP antibody (1:5000, Abcam, Cambridge, UK), rabbit anti-Iba1 antibody (1:10,000, FUJIFILM Wako Pure Chemical Corporation, Tokyo, Japan), or rabbit anti-iNOS antibody (1:5000, Abcam, Cambridge, UK), for 2 h at RT. The membranes were washed and incubated with a horseradish peroxidase-conjugated anti-rabbit antibody (1:2000, LI-COR Corporate, Lincoln, NE, USA) for 1 h at RT. Immunoreactive bands were detected using a western PREMIUM chemiluminescent substrate (LI-COR Corporate, NE, USA) and an LI-COR C-DiGit chemiluminescence Western blot scanner (LI-COR Corporate, NE, USA). These blots were then stripped using the Restore^TM^ Stripping Buffer (Thermo Fisher Scientific K. K., Tokyo, Japan) and incubated with a primary antibody against β-tubulin (ab52901, 1:5000, Abcam, Cambridge, UK), which was used as an internal loading control.

### 4.9. Data Analysis

The Western blot images and photo images were analyzed using Image J software (Version 1.54, NIH, Bethesda, MD, USA) and quantified. Statistical analysis was performed using StatView statistical software (Version 5.0, SAS Institute Inc., Cary, NC, USA). Differences were analyzed using analysis of variance, and significance was set at *p* < 0.05.

## Figures and Tables

**Table 1:** Blood chemistry data for both treated and untreated mice at various timepoints.

	TP (g/dL)	ALB (g/dL)	AST (IU/L)	ALT (IU/L)	ALP (IU/L)
Control	3.24 ± 0.29	1.49 ± 0.28	214.31 ± 3.94	10.83 ± 4.38	141.38 ± 18.15
3H	2.47 ± 0.21 *	1.69 ± 0.36	321.12 ± 28.47 *	18.68 ± 3.12	172.09 ± 10.29
24H	3.39 ± 0.28	1.74 ± 0.31	331.29 ± 29.11 *	53.22 ± 3.19 *	166.21 ± 14.34

Control, 3H, and 24H indicate the average blood chemistry values from the vehicle treatment group and the samples taken from animals 3 and 24 h following ammonia treatment, respectively. Data are expressed as mean ± standard deviation. TP—total protein; ALB—albumin; AST—aspartate aminotransferase; ALT—alanine aminotransferase; ALP—alkaline phosphatase; * *p* < 0.05, compared to the control.

## Data Availability

Not applicable.
